# Salpingitis. A rare cause of acute abdomen in a sexually inactive girl: a case report

**DOI:** 10.1186/1757-1626-1-326

**Published:** 2008-11-18

**Authors:** Mayke E van der Putten, Monique Engel, Gijs THJ van Well

**Affiliations:** 1 Maastricht University Medical Centre, Department of Pediatrics, P.O. Box 5800, 6202 AZ Maastricht, The Netherlands

## Abstract

Salpingitis is an acute inflammation of the fallopian tubes, most commonly caused by sexually transmitted micro-organisms in adolescent and adult women. It is rarely found in sexually inactive girls and generally the result of a blood-borne or genitourinary infection. In young girls without a history of consensual sexual contact, the possibility of sexual abuse should be considered.

Salpingitis usually presents as an acute abdomen. Appendicitis presents with almost the same symptoms as salpingitis. The diagnosis of salpingitis is often delayed until the presumed appendicitis is surgically explored.

We describe an 11-year-old girl with salpingitis caused by *Streptococcus pneumoniae*.

## Introduction

Salpingitis is an acute inflammation of the fallopian tubes, most commonly caused by sexually transmitted micro-organisms in adolescent and adult women.[1] It is very uncommon in premenarchal or sexually inactive girls.[2]

*Streptococcus pneumoniae *is a frequent cause of pneumonia, bacteremia, meningitis and otitis media in children. It is rarely the cause of primary peritonitis and it is even more unusual as cause of upper genital tract infections in premenarchal girls.[3]

We describe an 11-year-old girl with salpingitis caused by *Streptococcus pneumoniae*.

## Case presentation

A previously healthy 11-year-old Caucasian girl presented at the emergency department of a general hospital suffering from abdominal pain and fever. She also complained of nausea, vomiting and vaginal discharge was denied.

On physical examination, an ill looking girl with severe abdominal pain. She had a temperature of 39,6°C, heart rate of 116/minute and blood pressure of 123/66 mmHg. She had a painful abdomen on palpation with active muscular contractions and rebound tenderness. She did not have an irritated psoas muscle or pain at McBurney's point. On auscultation we found very little bowel movements. Vaginal discharge was not seen.

Laboratory results revealed a CRP of 307 mg/L (N < 10 mg/L) and leukocytes of 19,5 × 10^9^/L with 90% granulocytes, 8% band forms and 2% lymphocytes. Urine analysis showed 51–140 leukocytes/microliter, 11–20 erythrocytes/microliter and a negative nitrite.

The consulting pediatric surgeon performed an emergency laparoscopy because of suspected perforated appendicitis. Imaging was not performed. On laparoscopy, appendicitis or bowel perforation could not be confirmed but the right fallopian tube was coated with fibrin. Samples from the fibrin were taken for culture.

Post operatively amoxicillin-clavulanate was started intravenously at 100/10 mg/kg/day to cover Gram-positive, Gram-negative and anaerobic micro-organisms. Chlamydia coverage was not started because the patient did not have a history of sexual activity.

The next day *Streptococcus pneumoniae *was isolated from both the fallopian fibrin and the blood culture drawn upon admission. Antibiotic therapy was then switched to benzylpenicillin intravenously at 200.000 IU/kg/day and the patient was treated for ten days. She recovered quickly and was discharged from the hospital after ten days.

The possibility of sexual abuse was discussed explicitly but we did not find any indications. She denied having symptoms of pharyngitis or upper respiratory symptoms in the days prior to her admission.

Our patient will be followed up by her general practitioner and a gynecologist since infertility might be a complication of salpingitis.

## Discussion

Previously healthy children with acute abdominal pain, peritoneal irritation, fever and leukocytosis are often considered to have acute appendicitis.[3] This is the most frequent reason for emergency abdominal surgery in childhood.[3,5] Initially we also considered acute appendicitis to be the cause of our patient's symptoms. It was only after laparascopic surgery that this diagnosis was rejected because of an appendix sana and fibrin coating of the right fallopian tube. Fibrin coating on a fallopian tube is a characteristic appearance of salpingitis.[4,6]

Infertility and an increased chance of ectopic pregnancy are the most important long term complications of salpingitis.[1,4] The rate of infertility is approximately 15% after a first episode of salpingitis and increases to 50% after a third episode.[1,4] The most common cause of salpingitis in sexually active women is vaginal flora, introduced during sexual intercourse, or a sexually transmitted micro-organism from a sex partner, most commonly *Chlamydia trachomatis *and *Neisseria gonorroea*.[4] In young girls without a history of consensual sexual contact, the possibility of sexual abuse should always be considerd. In premenarchal and sexually inactive females other micro-organisms have been found to be the cause of pelvic inflammatory disease (PID). In case studies of premenarchal girls, *Neisseria gonorroea, Streptococcus pyogenes, Streptococcus pneumoniae, Escherichia coli *and *beta-hemolytic streptococcus of group F *have been described as causes of PID.[4] A cultured micro-organism in case of salpingitis may reveal the way in which salpingitis has been acquired. Salpingitis caused by *Streptococcus pneumoniae *develops after hematogenous or lymphatic spread from a primary focus of infection such as upper the respiratory tract but it may also result from transmural migration through an intact intestinal wall from the gut lumen. The infection can also ascend from the vagina through the fallopian tubes.[3,4]*Streptococcus pneumoniae *does not belong to the commensal vaginal flora.[3,7] Colonization can occur post partum, following an abortion, or after gynaecologic procedures.[3,1] In children, colonization can occur through inadequate hand hygiene, where micro-organisms are transferred from the upper respiratory tract to the urogenital tract.[3] Besides, one should also consider orogenital sexual contact in sexual abuse.[3,4] In 1993 Meis et al. described a 4-year-old girl with salpingitis after an abdominal trauma with retroperitoneal hematoma.[7]

Hematogenous distribution seems to be the cause of salpingitis in our patient, because of the positive blood culture with the same micro-organism found in the fibrin coating of the right fallopian tube. The days prior to her disease however, our patient did not have symptoms of upper respiratory tract infection or fever, indicating bacteremia. In our case we found no clues for sexual abuse.

## Patient's perspective

None.

## Abbreviations

See text.

## Consent

Written informed consent was obtained from the patient's parents for publication of this case report. A copy of the written consent is available for review by the Editor-in-Chief of this journal.

## Competing interests

The authors declare that they have no competing interests.

## Authors' contributions

All authors contributed to the acquisition of the case details and the analysis and interpretation of them. MEvdP wrote the first draft of the manuscript. ME and GThJvW revised the manuscript. All authors have given final approval of this version to be published.
